# LINC complex alterations in DMD and EDMD/CMT fibroblasts

**DOI:** 10.1016/j.ejcb.2012.03.003

**Published:** 2012-08

**Authors:** Surayya Taranum, Eva Vaylann, Peter Meinke, Sabu Abraham, Liu Yang, Sascha Neumann, Iakowos Karakesisoglou, Manfred Wehnert, Angelika A. Noegel

**Affiliations:** a Institute for Biochemistry I, Medical Faculty, University of Cologne, Joseph-Stelzmann-Str. 52, 50931 Cologne, Germany; b Center for Molecular Medicine Cologne (CMMC) and Cologne Excellence Cluster on Cellular Stress Responses in Aging-Associated Diseases (CECAD), Medical Faculty, University of Cologne, Joseph-Stelzmann-Str. 52, 50931 Cologne, Germany; c Ernst-Moritz-Arndt-University, Institute of Human Genetics, 17487 Greifswald, Germany; d Department of Biological Sciences, The School of Biological and Biomedical Sciences, The University of Durham, Durham, UK

**Keywords:** Nesprins, SUN2, Nuclear envelope, Laminopathy, EDMD, Muscular dystrophy, SUN2 proteome

## Abstract

Emery-Dreifuss muscular dystrophy (EDMD) is a late onset-disease characterized by skeletal muscle wasting and heart defects with associated risk of sudden death. The autosomal dominant form of the disease is caused by mutations in the *LMNA* gene encoding LaminA and C, the X-linked form results from mutations in the gene encoding the inner nuclear membrane protein Emerin (*STA*). Both Emerin and LaminA/C interact with the nuclear envelope proteins Nesprin-1 and -2 and mutations in genes encoding C-terminal isoforms of Nesprin-1 and -2 have also been implicated in EDMD. Here we analyse primary fibroblasts from patients affected by either Duchenne muscular dystrophy (DMD) or Emery-Dreifuss muscular dystrophy/Charcot-Marie-Tooth syndrome (EDMD/CMT) that in addition to the disease causing mutations harbour mutations in the Nesprin-1 gene and in the SUN1 and SUN2 gene, respectively. SUN proteins together with the Nesprins form the core of the LINC complex which connects the nucleus with the cytoskeleton. The mutations are accompanied by changes in cell adhesion, cell migration, senescence, and stress response, as well as in nuclear shape and nuclear envelope composition which are changes characteristic for laminopathies. Our results point to a potential influence of mutations in components of the LINC complex on the clinical outcome and the molecular pathology in the patients.

## Introduction

The nuclear envelope (NE) forms an interface between the cytoplasm and the nuclear interior during interphase. It is composed of the outer and inner nuclear membranes (ONM and INM, respectively), the nuclear lamina and the nuclear pore complexes (NPCs). The ONM is continuous with the endoplasmic reticulum (ER) and connects with the INM at the NPCs, the INM associates with the nuclear lamina and with chromatin (Mellad et al., 2011; Starr and Fridolfsson, 2010). The NE proteome comprises several proteins that perform critical functions in the maintenance of nuclear architecture, positioning and migration. Mutations in these proteins lead to a variety of diseases, collectively called laminopathies, including partial lipodystrophy, mandibulolacral dysplasia (MAD), dilated cardiomyopathy, autosomal-dominant limb-girdle muscular dystrophy type 1B (LGMD1B), Charcot-Marie-Tooth neuropathy type 2B1 (CMT2B1), restrictive dermopathy, Hutchinson-Gilford progeria, Werner's syndrome and Emery-Dreifuss muscular dystrophy (EDMD) (Capell and Collins, 2006). EDMD is typically characterised by the clinical triad of (1) early contractures of the Achilles tendons, elbows and postcervical muscles (with subsequent limitation of neck flexion, but later forward flexion of the entire spine becomes limited); (2) progressive skeletal muscle weakness and wasting with a humero-peroneal predominance at the onset of the disease (i.e. proximal in the upper limbs and distal in the lower limbs) and (3) a life threatening cardiac disease where conduction defects coexist with ventricular and supraventricular arrhythmias, chamber dilation and heart failure.

Charcot-Marie-Tooth disease (CMT) constitutes a clinically and genetically heterogeneous group of hereditary motor and sensory neuropathies. On the basis of electrophysiological criteria, CMT can be divided into two major types: type 1, the demyelinating forms, characterised by a motor median nerve conduction velocity less than 38 m/s; and type 2, the axonal form, with a normal or slightly reduced nerve conduction velocity (The UMD-LMNA database: www.umd.be/LMNA).

To date, mutations in *LMNA*, *STA* and the Nesprin-1 and -2 genes *SYNE1* and *SYNE2* have been associated with EDMD. Mutations in the *LMNA* gene encoding the intermediate filament proteins LaminA and C of the nuclear lamina cause the autosomal dominant form of the disease while mutations in the *STA* gene encoding the INM protein Emerin cause the X-linked form (Bione et al., 1994; Bonne et al., 1999; Manilal et al., 1996; Nagano et al., 2006). SUN proteins like SUN1 and SUN2 and Nesprin-1 and- 2 are direct interaction partners of Emerin and LaminA/C and are candidates for those cases of EDMD which do not involve Emerin or LaminA/C (Zhang et al., 2007).

SUN1 and 2 are widely expressed type II INM proteins containing at least one transmembrane domain and a conserved C-terminal SUN domain located within the lumen of the NE. Through this domain they can recruit KASH (Klarsicht, ANC-1, Syne homology) domain proteins to the NE forming the LINC complex (**li**nker of **n**ucleoskeleton and **c**ytoskeleton) that provides a connection between the nucleoplasm and the cytoplasm and links it to the cytoskeleton (Crisp et al., 2006; Haque et al., 2006; Padmakumar et al., 2005; Starr and Han, 2002; Starr et al., 2001; Stewart et al., 2007; Stewart-Hutchinson et al., 2008).

The KASH domain containing Nesprins (**N**uclear **e**nvelope **sp**ectrin **r**epeat prote**ins**) are spectrin repeat containing type II transmembrane proteins that localise to the nuclear envelope and are encoded by independent genes. Several isoforms exist that are generated by the use of internal initiation sites or by alternative splicing and vary in domain composition. The largest isoforms of Nesprin-1 and -2 (∼1000 kDa and ∼800 kDa, respectively) contain an N-terminal actin binding domain, a long rod domain with several spectrin repeats and a C-terminal KASH domain which tethers them to the nuclear membrane (Apel et al., 2000; Mislow et al., 2002a; Padmakumar et al., 2004; Simpson and Roberts, 2008; Zhen et al., 2002). Here we analysed components of the LINC complex in fibroblasts from a healthy donor individual and patients suffering from Duchenne muscular dystrophy (DMD) and Emery-Dreifuss muscular dystrophy/Charcot-Marie-Tooth syndrome (EDMD/CMT). Sequence analysis revealed a mutation in the *DMD* gene in the case of the DMD patient, whereas for the EDMD/CMT the underlying mutation could not be identified. Furthermore, the patients harboured additional mutations in components of the LINC complex which initiated an analysis of NE components with particular emphasis on SUN2 and led to the establishment of a SUN2 proteome for the EDMD/CMT fibroblasts which might deliver novel insights into the underlying pathomechanism of the disease.

## Materials and methods

### Cell culture

Human control and patient primary fibroblasts of a male (G-11235) and a female patient (G-11847) suffering from Duchenne muscular dystrophy (DMD) and Emery-Dreifuss and Charcot-Marie-Tooth syndrome (EDMD/CMT), respectively, were cultured in Eagle's DMEM (Gibco) supplemented with 20% FBS, 1 mM glutamine, 1% penicillin/streptomycin and 7.5% sodium bicarbonate at 37 °C and 5% CO_2_. Patients and mutation analysis have been described in Zhang et al. (2007). Cells were used between passage 4 and passage 16 as indicated. DMD myofibroblasts were obtained from the Muscle Tissue Culture Collection, Friedrich-Baur-Institute, LMU München.

### Transfection experiments

A mutation at position c.278A>C of SUN1 which leads to an amino acid exchange p.Q93P was introduced into plasmid GFP-hSun1 (Lu et al., 2008). Sun2-V5-His (Lu et al., 2008) was used for introduction of the mutation at position c.97A>G which leads to an amino acid exchange p.T33A in SUN2. A site directed mutagenesis kit (Promega) was used. The mutation was confirmed by DNA sequencing. HaCaT cells were transfected using the Amaxa cell line Nucleofector^®^ kit V (Lonza) according to the manufacturer's instructions. Cells were analysed by confocal microscopy for the presence and distribution of nuclear envelope markers.

### Immunofluorescence analysis

Cultured cells grown on coverslips were fixed in 3% paraformaldehyde in PBS for 10 min followed by permeabilisation with 0.5% Triton X-100 for 5 min for Nesprin-2 staining. Alternatively, cells were fixed in cold methanol (−20 °C) for 5 min for Nesprin-1 staining. The following antibodies were used: mouse monoclonal anti-Nesprin-2 mAb K20-478 (Zhen et al., 2002), affinity-purified rabbit anti-Nesprin-1 antibody SpecII (this study), rabbit polyclonal Nesprin-2 pAbK1 (Libotte et al., 2005), rabbit anti-LaminB1 polyclonal antibodies (Abcam), mouse monoclonal anti-Emerin (Abcam), rabbit polyclonal anti-LaminA/C (Santa Cruz), polyclonal antibodies specific for pericentrin (Abcam), mouse monoclonal anti vinculin antibody (Sigma), mouse monoclonal anti-V5 antibody (Invitrogen). The secondary antibodies used were conjugated with Alexa Fluor 488 (Molecular Probes), Cy3, TRITC and FITC (Sigma). The samples were counterstained with DAPI (Sigma) and mounted in gelvatol. Samples were analysed using confocal laser scanning microscopy (TCS-SP5, Leica). In general, cells between passages 8 and 16 were used for immunofluorescence.

### Antibody generation

For the investigation of Nesprin-1 polyclonal antibodies (SpecII) directed against the C-terminus of human Nesprin-1 were generated. The last two spectrin repeats (residues 8394–8608) of Nesprin-1 were expressed as GST-fusion protein (GST-SpecII) and used for immunisation of rabbits. Nesprin-1 specific antibodies were purified by affinity chromatography. For this, the Nesprin-1 polypeptide of GST-SpecII was cleaved from GST with thrombin, dialysed and coupled to CNBr-activated sepharose. The purified antibodies were highly specific for Nesprin-1. They reacted with GFP fusion proteins containing the Nesprin-1 C-terminus but not with GFP-fusions of the corresponding Nesprin-2 C-terminal sequences. They were used for immunofluorescence and Western blot analysis. Monoclonal antibodies specific for SUN2 were generated against an N-terminal polypeptide of human SUN2 (Q9UH99), SUN2Nt, encompassing residues 1–138 and which is located in the nucleoplasm. The ∼15 kDa polypeptide has a pI of 9.4. It was expressed as GST-fusion in *E. coli* XL1-Blue. After thrombin cleavage the polypeptide was used for immunisation of mice. mAb K80-207 was used in this work. The specificity of the antibodies was confirmed in experiments with V5-His-tagged SUN2 (Lu et al., 2008).

### Western blotting

Cells were grown to confluency, washed twice with ice cold PBS and resuspended in modified radio-immunoprecipitation (RIPA) lysis buffer (50 mM Tris–HCl, pH 7.5, 150 mM NaCl, 1% NP-40, 0.5% Na desoxycholate, 0.1% SDS, 1 mM dithiothreitol, 1 mM benzamidine, 1 mM PMSF, 1 mM EGTA, 10 μg/ml aprotinin and 10 μg/ml leupeptin). Cell suspensions were passed through a 0.45 μm needle 10 times and incubated for 15 min on ice, followed by sonication. The lysates were cleared by centrifugation at 10,000 rpm for 5 min at 4 °C. The samples were resuspended in 5× SDS sample buffer and heated at 98 °C for 5–10 min. For Western blot analysis, proteins were resolved on 10%, 12% SDS- or 3–15% gradient SDS polyacrylamide gels and transferred to PVDF membrane (Pall Corporation) over 24–72 h. The blots were probed with primary antibodies, washed with TBST (10 mM Tris–HCl, pH 8.0, 150 mM NaCl and 0.05% Tween-20) several times and incubated with horseradish peroxidase-coupled secondary antibody (Dianova, Germany). The blots were washed again with TBST and visualised using enhanced chemiluminescence (ECL) system (Luminol, p-Cumaric acid, Sigma–Aldrich, Steinheim, Germany). For control α-tubulin specific antibody mAb WA3 provided by Dr. U. Euteneuer was used.

### Heat stress experiments

Normal and patient fibroblasts were trypsinised two days before the experiment and plated on 12 mm glass coverslips and cultured at 37 °C. For heat stress, cells were placed in an incubator set at 45 °C for 30 min and fixed immediately after treatment with cold methanol at −20 °C for 5 min and incubated with anti-LaminB1 rabbit polyclonal antibodies or monoclonal antibody 4G5 (Abcam) against emerin to detect the nuclear envelope and pericentrin specific polyclonal antibodies to detect the centrosome. Untreated normal and patient fibroblasts were used as control.

### GST pull-down assay

Recombinant GST-SUN2Nt polypeptide expression was induced in *E. coli* strain BL21 (0.5 mM IPTG, 4 h, 37 °C). Cells were lysed as described (Libotte et al., 2005) and the proteins were isolated from the supernatant by incubating with glutathione agarose beads (4 h, 4 °C). Glutathione agarose beads coupled with GST-SUN2Nt were washed five times with PBS (500 g, 4 °C, 1 min) before incubating with the according human fibroblast total cell lysates (100,000 g supernatant) at 4 °C for 6 h. Beads were washed three times with PBS (500 g, 4 °C, 1 min) and heated in SDS sample buffer (95 °C, 5 min). Samples were analysed using 12% SDS polyacrylamide gels and stained with Coomassie Brilliant Blue. Protein bands of interest were excised from the gels and subjected to LCMS analysis. The experiment was carried out twice. For the analysis we used control fibroblasts and EDMD/CMT fibroblasts. The EDMD/CMT fibroblasts were taken at passage 6 and 8, respectively.

### Senescence-associated β-galactosidase assays

Cells were seeded on cover slips; the next day cover slips were washed with PBS and fixed with 3% PFA (5 min, RT). Cells were washed twice with PBS and incubated at 37 °C with freshly prepared senescence-associated β-Gal (SA-β-Gal) staining solution (van der Loo et al., 1998). Examination for staining was done after 4–8 h under bright field microscopy at 40× magnification.

### RNA isolation and cDNA generation for quantitative RT-PCR analysis

Total RNA was extracted from cells grown in a monolayer in cell culture dishes with a kit following the instructions of the supplier (Promega, Heidelberg, Germany). First-strand cDNA synthesis was performed using M-MLV reverse transcriptase RNase H Minus-kit from Promega. To evaluate the gene expression level of SUN2 by quantitative RT-PCR in the patient fibroblast in comparison to the control fibroblasts, total RNA was isolated from control fibroblasts at passage 8 and 22, EDMD/CMT fibroblasts at passage 4 and 16, DMD fibroblasts at passage 6 and 8. For normalisation GAPDH expression was used.

### Focal adhesion assay

Trypsinised cells were seeded on coverslips in culture dishes with an initial cell number of 1 × 10^3^ and subjected to immunofluorescence as described above by staining for vinculin. Analysis was carried out with a confocal laser scanning microscope TCS-SP5 (Leica) equipped with TCSNT software. The individual immunofluorescences shown in Fig. 4A have the same magnification (scale bar, 76.4 μm) and were taken in the same *z*-plane so that the spreading of focal adhesions on the surface of the coverslip is comparable. LAS-AF Lite Application Suite software from Leica was used to quantify the spreading area in μm^2^.

### Wound healing assay

Control and patient cells were grown on 10 cm tissue culture plates until nearly confluent. Before experimentation, the cells were serum-starved overnight. The next day the medium was replaced with serum-supplemented normal growth medium. A scratch was made in the monolayer with a 200 μl tip and the rate of cell migration into the wounded area was monitored using bright field microscopy. Images were captured at various time points and the distance migrated by the cells was calculated by measuring the open wound area. Experiments were repeated at least thrice for each cell type.

## Results

### Case report of Duchenne muscular dystrophy (DMD), and Emery-Dreifuss muscular dystrophy/Charcot-Marie-Tooth syndrome (EDMD/CMT) patients

We used primary fibroblasts from a patient (G-11235) suffering from a muscular dystrophy compatible with a clinical phenotype of Emery-Dreifuss muscular dystrophy (EDMD). Mutational analysis was negative in the genes known to be associated to EDMD including *STA, LMNA FHL1*. We therefore extended the analysis to components of the LINC complex and sequenced in particular coding exons of Nesprin-1α1, Nesprin-1α2, Nesprin-2α1, Nesprin-2α2, Nesprin-2β1 and Nesprin-2β2, *SUN1* and *SUN2*. The *DMD* gene was sequenced as well. This revealed the presence of a nonsense mutation c.3409C>T in the *DMD* gene leading to a truncated dystrophin p.E1137X. The p.E1137X mutation is located in spectrin repeat 7 of the protein (Legrand et al., 2011) (NCBI AAA53189.1). Based on this, patient G-11235 should phenotypically be considered as Duchenne muscular dystrophy (DMD). In addition, patient G-11235 had a 29A>G mutation in the 5′UTR of the Nesprin-1α2 isoform which was not present in a reference population (386 alleles) suggesting a transcriptional mis-regulation of this Nesprin-1 isoform. Nesprin-1α is a C-terminal isoform of Nesprin-1 and is primarily expressed in skeletal and cardiac muscle. It contains a transmembrane domain and several spectrin repeats but lacks the actin binding domain (Apel et al., 2000; Mislow et al., 2002a). The patient further harboured a mutation in *SUN1* (c.278A>C, p.Q93P).

Patient G-11847 suffers from a neuromuscular disorder showing clinical signs of EDMD and Charcot-Marie-Tooth syndrome (CMT). She was found to be heterozygous for a mutation p.N323H in a spectrin repeat of Nesprin-1α1. The patient inherited this mutation from her clinically healthy Asian mother. The p.N323H mutation was not found in Caucasians (254 alleles) but occurred in 4% of an Asian reference population (150 alleles). No primarily pathogenic mutation for the disease in G-11847 could be found. G-11847 also carried a mutation in *SUN2* at position c.97A>G which leads to an amino acid exchange p.T33A. This exchange represents a rare variant which appears to occur only in Asian populations (2/252 chromosomes). At the protein level, the mutation is located in the highly conserved N-terminal region of SUN2.

Obviously, the Nesprin-1 mutations found in patients G-11235 and 11847, respectively, are not primarily pathogenic. However, to find out a possible contribution of those Nesprin-1 mutations to the clinical outcome we carried out investigations on the cellular level to probe for alterations that are associated with diseases caused by mutations in components of the NE.

### Patient fibroblasts show nuclear shape defects and have alterations in components of the nuclear envelope

**Fig. 1:**
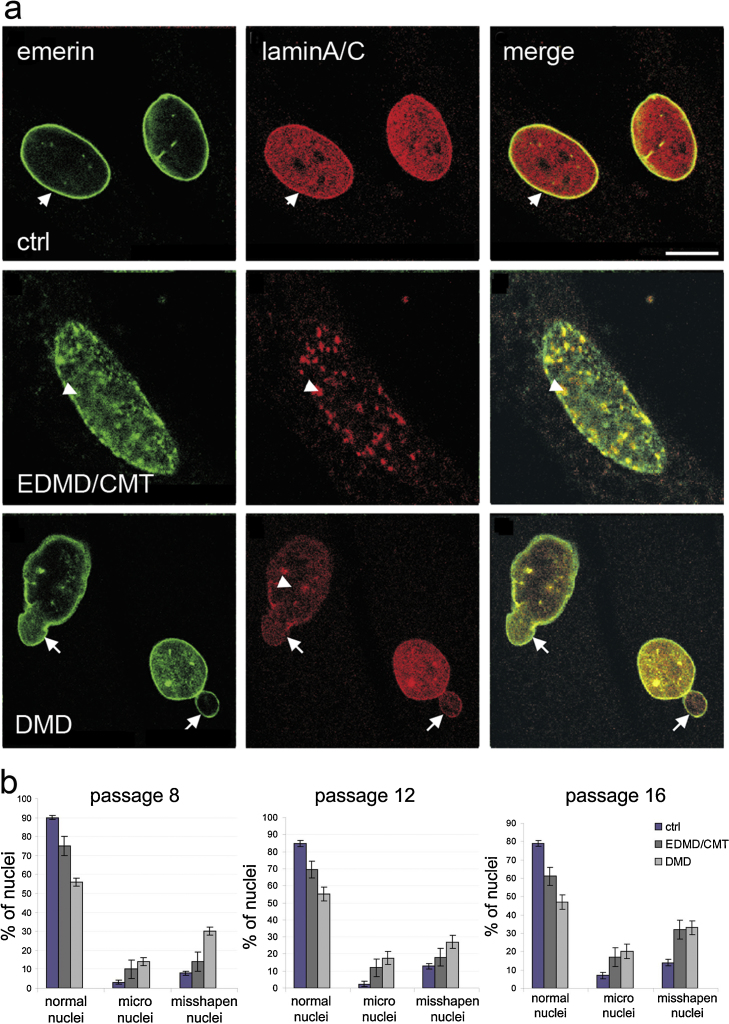
Nuclear defects in DMD and EDMD/CMT fibroblasts. (a) Control (ctrl) and patient fibroblasts (EDMD/CMT, DMD) were stained for Emerin (left panel) and LaminA (middle panel). Merge, right panel. Arrows in ctrl, Emerin and LaminA/C colocalisation; arrowheads (EDMD/CMT), colocalisation of Emerin and LaminA/C in nuclear patches; DMD, arrow, nuclear lobulation and bleb; DMD, middle panel, arrow head points to a nuclear patch. Alexa Fluor 568 and 488 conjugated secondary antibodies were used and DAPI for nuclear staining. Bar, 10 μm. (b) The statistical analysis shows the increase of micronuclei and misshapen nuclei at increased passages. As misshapen we defined nuclei which did not have an ovoid or round shape. One hundred nuclei per control and patient cells were evaluated at passage 8, 12 and 16 each. The error bars indicate standard deviations. The experiment was carried out twice.

Nuclei of control cells generally have a round or ovoid shape. In the patient fibroblasts we noted a variety of nuclear shape defects including folds, lobulations, blebs and micronuclei (Fig. 1a). Quantification showed that the number of cells with nuclear defects was increased compared to control cells (Fig. 1b). The DMD patient cells exhibited the most significant nuclear shape alterations. At an average, 30% of DMD cells showed misshapen nuclei with a slight increase from passage 8 to passage 16. In contrast, EDMD/CMT fibroblasts exhibited 15 and 19% misshapen cells in passage 8 and passage 12, respectively, and at passage 16 an increase to 32% was observed. The relation between micronuclei and misshapen cells correlated and remained almost constant (Fig. 1b).

When probing NE components in immunofluorescence analyses we found that nuclei of control cells displayed homogenous distribution of Emerin and LaminA/C at the nuclear periphery. LaminA/C showed in addition an even distribution throughout the nucleus (Fig. 1a, arrows). In EDMD/CMT patient fibroblasts, the peripheral distribution of Emerin was altered to a patchy staining of the nucleus which was also observed for LaminA/C (Fig. 1a). In fact, Emerin and LaminA/C colocalise frequently in these patches (Fig. 1a, arrowheads). In the DMD cells the nuclear rim staining was retained for Emerin and Lamin and a localisation of Emerin and LaminA/C in blebs and protrusions was observed in dysmorphic nuclei (Fig. 1a, arrow). Further, a fraction of fibroblasts also displayed reduced LaminA/C staining. However, in the nuclear blebs depleted for LaminA/C, Emerin staining was still observed (Fig. 1a, arrows).

**Fig. 2:**
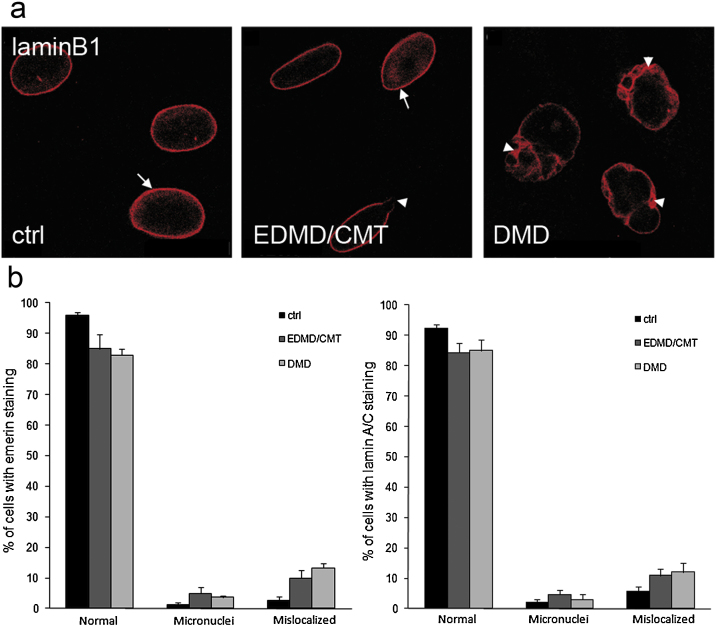
The distribution of nuclear envelope proteins is altered in patient fibroblasts. (a) LaminB1 was stained using anti-LaminB1 as first antibodies; Alexa Fluor 568 as conjugated secondary antibodies; DAPI for nuclear staining. Arrows and arrowheads indicate the defects described in the text. Bar, 10 μm. (b) Statistical analysis of observed defects in Emerin and LaminA/C localisation (see Fig. 1a for staining) was carried out for each mutation. Nearly 300 nuclei were counted for each sample analysed. Error bars indicate standard deviations.

**Fig. 3:**
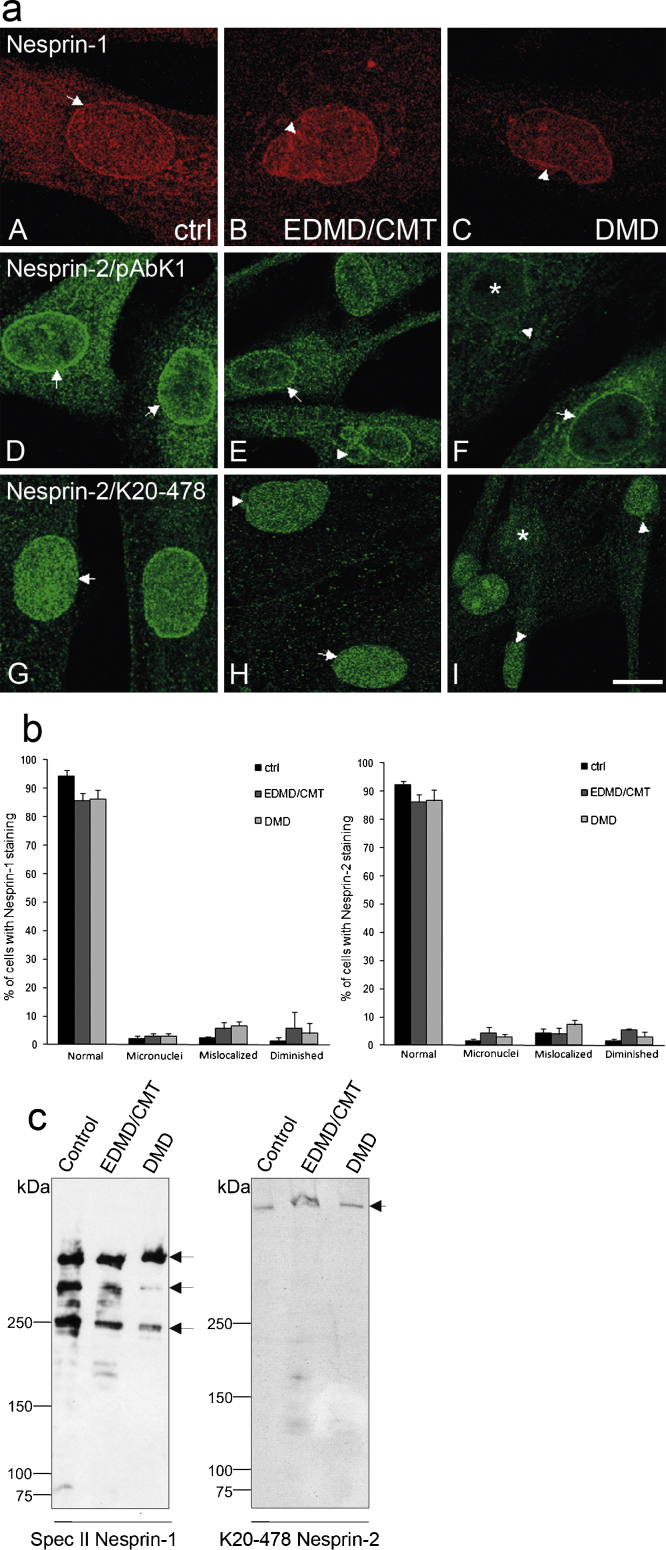
Nesprin-1 and -2 localisation is altered in fibroblasts from DMD and EDMD/CMT patients. (a) Control, EDMD/CMT and DMD fibroblasts were stained for Nesprin1 using anti-Nesprin-1 SpecII and Nesprin-2 using pAbK1 and mAb K20-478 specific for the C- and N-terminus of Nesprin-2, respectively, as first antibodies; Alexa Fluor 568 and 488 as secondary antibodies; DAPI for nuclear staining. Arrows and arrowheads indicate the defects described in the text. Bar, 10 μm. (b) Statistical evaluation of the observed defects. Approximately 300 nuclei were evaluated for each mutation. (c) Presence of Nesprin-1 isoforms and of Nesprin-2 Giant in control and patient fibroblasts. Cell lysates were separated on SDS gradient gels (3–15% acrylamide) and probed with SpecII antibodies specific for Nesprin-1 and mAb K20-478 recognising the ∼800 kDa Nesprin-2 Giant. Equal loading was assessed by Ponceau S staining of the blots.

LaminB1 antibodies showed the protein at the nuclear envelope in control fibroblasts (Fig. 2a). In EDMD/CMT fibroblasts the staining was discontinuous in nuclei that had blebs and blebs did not show significant LaminB1 labelling (Fig. 2a, arrowhead). Nuclei with normal shape showed the expected nuclear distribution of LaminB1 (Fig. 2a, arrow). In DMD fibroblasts the nuclear rim staining was not homogenous in misshapen nuclei (Fig. 2a, arrowhead). This aspect was observed in nuclei from cells at different passage numbers. Another notable aspect was the elongated appearance of nuclei in a fraction of cells. A statistical analysis was carried out to quantify the observations which confirmed increased numbers of patient cells exhibiting abnormalities (Figs. 2b and 3b).

When we tested cells from patients suffering from Becker muscular dystrophy for nuclear shape and distribution of NE proteins we observed normal shaped nuclei and a regular staining for the NE proteins (data not shown). This suggests that nuclear shape alterations are not generally associated with muscular dystrophy mutation. The patients harboured deletions of exons 2–11, exons 3–7 and 52–60, respectively, in the *DMD* gene.

### Nesprin distribution and abundance is altered in control and patient fibroblasts

Based on the findings that the patients have mutations in Nesprin-1 we investigated the localisation of Nesprin-1 and 2 using polyclonal antibodies directed against the C-terminus of Nesprin-1 and -2 (SpecII and pAbK1, respectively) and mAb K20-478 directed against the N-terminus of Nesprin-2 to detect Nesprin-2 Giant (Padmakumar et al., 2005; Zhen et al., 2002). Control cells exhibited clear nuclear envelope localisation for both Nesprin-1 and -2 with C-terminus specific antibodies (Fig. 3a, A, D, arrow). Nuclear staining was also observed for Nesprin-2 Giant with mAb K20-478 (Fig. 3a, G). EDMD/CMT and DMD cells showed nuclear envelope staining for Nesprin-1 (Fig. 3a, B, C). In irregular shaped nuclei we saw also distribution into folds and pleats (Fig. 3a, B, arrowhead). pAbK1 recognised Nesprin-2 at the nuclear envelope, and nuclei with misshapen morphology showed Nesprin-2 distribution into blebs (Fig. 3a, E, arrowhead). By contrast, envelope staining with mAb K20-478 was not pronounced in both patient fibroblasts. Instead, Nesprin-2 Giant staining was uniform throughout the nucleus in normal shaped nuclei and in nuclei with altered shape. A fraction of nuclei in the DMD cells showed diminished nuclear staining of Nesprin-2 Giant with concomitant defects in the nuclear shape (Fig. 3a, I, asterisk). A statistical evaluation revealed the increase of micronuclei, mislocalisation of Nesprins and reduced staining in the patient cells (Fig. 3b).

To analyse the isoform expression patterns of Nesprin-1 and -2, we performed Western blot analysis with pAb SpecII for Nesprin-1 and mAb K20-478 specific for Nesprin-2 (Fig. 3c). For Nesprin-1 we observed a similar pattern in control and patient cell lysates and detected proteins of ∼250 kDa, 400 and 600 kDa (Fig. 3c, arrows). However the intensity of the 250 and 400 kDa protein bands was reduced. This was most prominent in the DMD fibroblasts. Furthermore, proteins of lower molecular masses corresponding to C-terminal Nesprin-1 isoforms were not visible in the patient lysates. By contrast, Nesprin-2 Giant levels were comparable to the control in both patient cell lines.

### SUN2 gene expression is down-regulated during passaging of patient cells

**Fig. 4:**
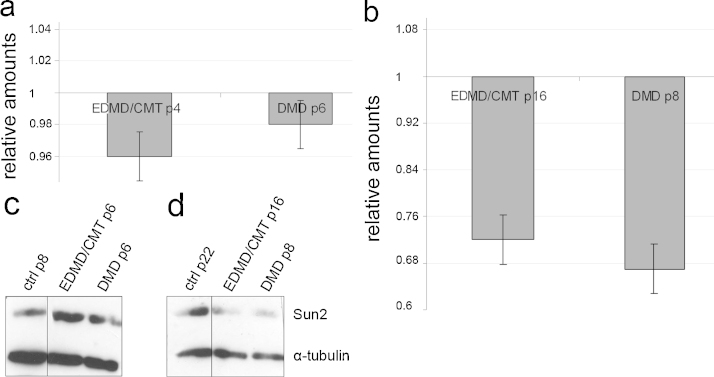
SUN2 transcript and protein levels in control and patient fibroblasts at different passages. (a) Evaluation of Sun2 mRNA by qRT-PCR. The SUN2 mRNA levels in control fibroblasts at passage 8 were set at 1.0, EDMD/CMT fibroblasts at passage 4 (p4), DMD fibroblasts at passage 6 (p6). Relative amounts as compared to control fibroblasts are shown. (b) SUN2 transcript levels at higher passages. Control fibroblasts at passage 22 were taken for reference. EDMD/CMT fibroblasts at passage 16 (p16), DMD fibroblasts at passage 8 (p8). (c, d) SUN2 protein levels as determined by immunoblot analysis of lysates from control and patient fibroblasts at different passages as indicated. For loading control the blots were probed for α-tubulin. mAb K80-207-11 was used for SUN2 detection, mAb WA3 for α-tubulin detection.

SUN proteins are essential components of the LINC complex. In humans there exist several SUN isoforms with SUN2 being the ubiquitously expressed one. In the following studies we focused on SUN2 as the gene carries a mutation in the EDMD/CMT fibroblasts. SUN2 specific antibodies showed a rim like staining pattern in DMD and EDMD/CMT fibroblasts comparable to wild type fibroblasts (data not shown). When we probed SUN2 expression at various passages we found that at lower passages (p6, p4, respectively) SUN2 expression is comparable to wild type fibroblasts as analysed by qRT-PCR, whereas at higher passages (p8, p16, respectively) the expression level of SUN2 is remarkably decreased (Fig. 4a, b). Immunoblot analysis of control and patient fibroblasts with the SUN2 specific mAb K80-207-11 confirmed the results obtained for the mRNA at the protein level revealing a decrease in EDMD/CMT cell lysates at higher passages (passage 6 vs passage 8 or 16, respectively) (Fig. 4c, d).

The diminished SUN2 transcript levels in senescent patient cells might be a consequence of the mutations in SUN1 and SUN2, respectively, leading to a weakening of the interactions of the LINC complex or through perturbed mechanotransduction and subsequent aberrant signalling pathways which in turn can affect the levels of cellular and extracellular components.

### The SUN2 proteome of EDMD/CMT fibroblast cells differs from that of control fibroblast cells

**Fig. 5:**
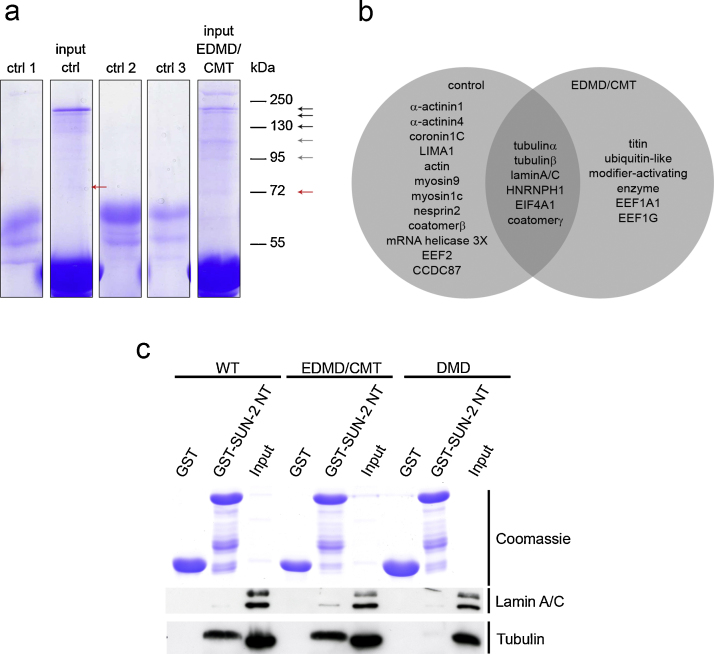
SUN2 proteome of control and EDMD/CMT fibroblasts. (a) Coomassie Blue stained SDS polyacrylamide gel (12% acrylamide) showing proteins from pull down assays using total control and EDMD/CMT cell lysates and GST-SUN2Nt as bait. Controls; ctrl1, GST-SUN2Nt beads incubated with PBS, ctrl2 and ctrl3, GST beads incubated with cell lysate from control fibroblasts (ctrl2) and EDMD/CMT cell lysate (ctrl3). Input ctrl and input EDMD/CMT show the proteins that were precipitated using GST-SUN2Nt coated beads from the corresponding lysates. Arrows point to the analysed gel areas. Black arrows, protein bands present in both samples; grey arrows, diminished in control or patient cell lysate; red arrows, only found in control or patient cell lysate. (b) Graphical representation of Sun2 binding partners in control and EDMD/CMT fibroblasts. The experiment was carried out twice. Only proteins that were detected in both analyses are shown. (c) Pull down experiments using GST (control) and GST-SUN2NT bound to Sepharose beads to precipitate LaminA/C and Tubulin from control (WT) and patient fibroblast lysates (EDMD/CMT and DMD). The precipitated proteins were detected with appropriate antibodies.

In independent work we have generated a SUN2 proteome for HaCaT cells and identified proteins belonging to several functional classes (Vaylann and Noegel, unpublished). Here we followed up and extended this work and carried out pull down experiments with total lysates from control and EDMD/CMT fibroblasts using the N-terminus of SUN2, GST-SUN2Nt, as bait (Fig. 5a). This approach might help to identify possible differences in protein interaction partners of SUN2 in control and patient fibroblasts. The analysis was done by separating the protein complexes by SDS-PAGE followed by LC–MS (liquid chromatography–mass spectrometry). The identified proteins have roles in gene regulatory events (A), in RNA-processing complexes (B), in architectural complexes (C) and in signalling (D) (Table 1). Fig. 5b summarises the data and illustrates the distribution of the proteins in control cell lysates and EDMD/CMT cell lysates. Some proteins are present in both lysates (Fig. 5b, intersection). Only one protein of category A, the coiled coil domain containing protein 87 (CCDC87) which has homology to MAP65 (microtubule associated protein 65) in its C-terminus, was shared by the proteomes of both cells. Equal numbers of proteins participating in RNA-processing were detected in both lysates. In control cell lysates significantly more proteins contributing to cell architecture and signalling events were found as compared to the EDMD/CMT cell lysate.

Proteins missing from the SUN2 proteome of EDMD/CMT cells were mainly actin cytoskeleton related proteins such as actin, myosin1C and myosin9. Notably, Nesprin-2 was only found in control cell lysates, LaminA/C was identified in both lysates (Table 1 and Fig. 5b). The Nesprin-2 peptide identified in control cells matches to a sequence in the last spectrin repeat which is present in the Nesprin-2 isoforms Nesprin-2 Giant, Nesprin-2α2 and Nesprin-2α1. We verified some interactions by repeating the pull down and probing the presence of tubulin and LaminA/C in the precipitate with antibodies (Fig. 5c). In this experiment we also included a lysate from cells of the DMD patient. LaminA/C was pulled down in nearly comparable amounts from all cell lysates, whereas for tubulin the signal in the DMD pull down was strongly reduced in comparison to control (WT) and EDMD/CMT cell lysates. GST was used for control.

### Expression of mutant SUN1 and SUN2 proteins in HaCaT cells

**Fig. 6:**
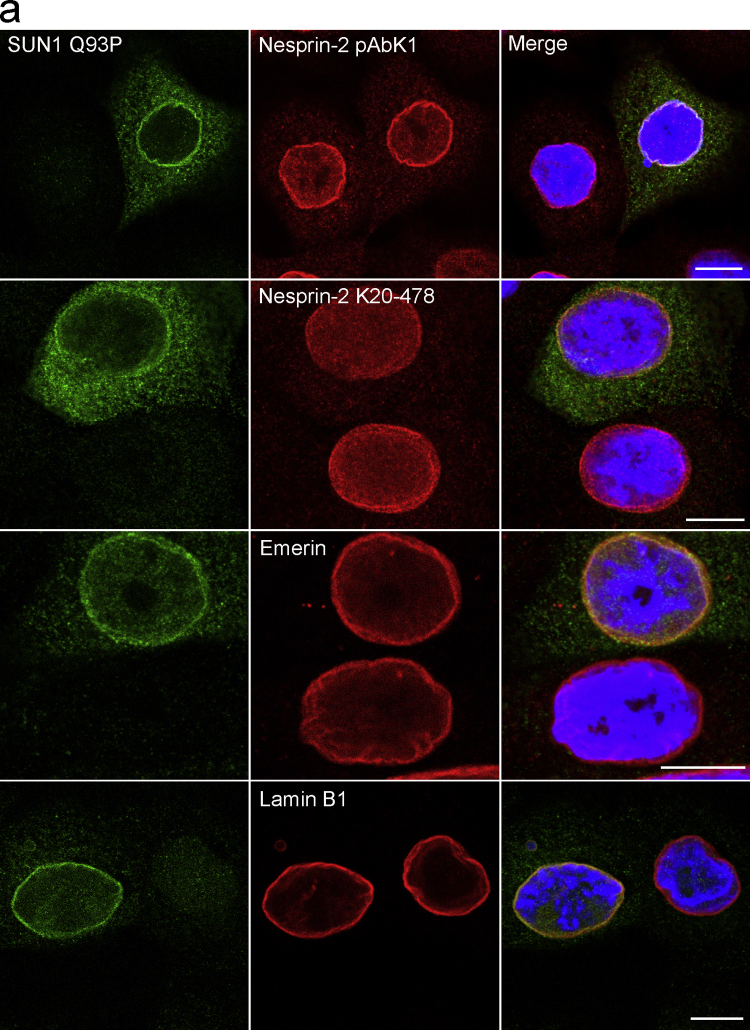

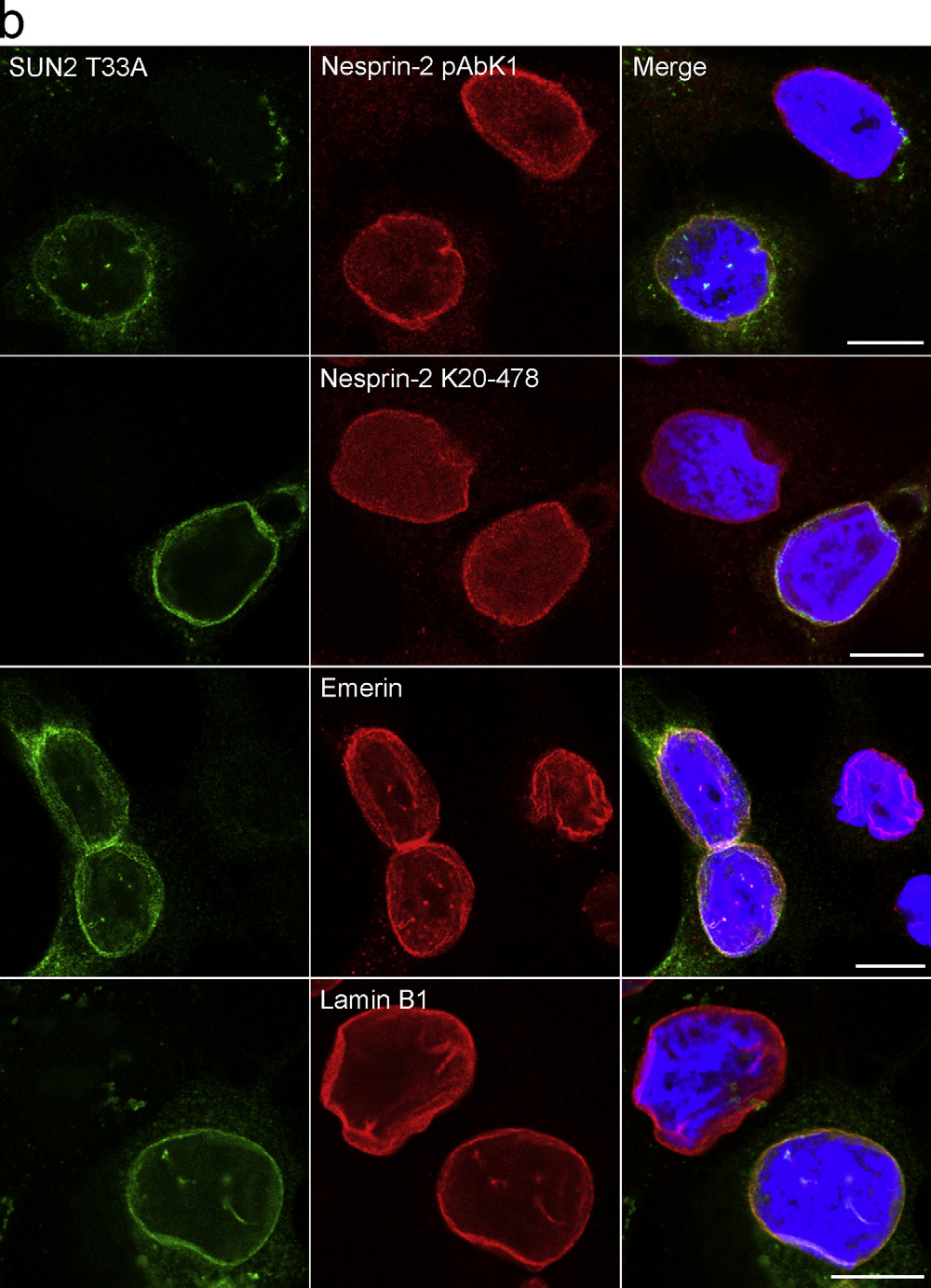
Expression of mutant SUN1 and SUN2 in HaCaT cells. (a) HaCaT cells expressing GFP-SUN1 carrying the mutation Q93P were analysed for Nesprin-2, Emerin and LaminB1 distribution using the corresponding antibodies. In (b) HaCaT cells expressing SUN2-V5-His carrying the mutation T33A were analysed. SUN2-V5-His was recognised using a V5-specific antibody. Bar, 10 μm.

Since SUN proteins are crucial components of the LINC complex we decided to probe the impact of the mutations found in the patients on this complex. The corresponding mutations were introduced into the respective cDNAs by site directed mutagenesis and the mutant proteins expressed in HaCaT cells. For the mutagenesis plasmids GFP-hSun1 and Sun2-V5-His (Lu et al., 2008) were used. Following transfection the presence of nuclear envelope components was probed by immunofluorescence analysis. We observed no changes at this level of analysis with regard to abundance and distribution of Nesprin-2, Emerin and LaminB1 (Fig. 6a, b). The low transfection efficiency prevented us from carrying out biochemical analysis which could give information on the stability of the LINC complex.

### The nucleus-centrosome distance is increased in EDMD/CMT and DMD fibroblasts

**Fig. 7:**
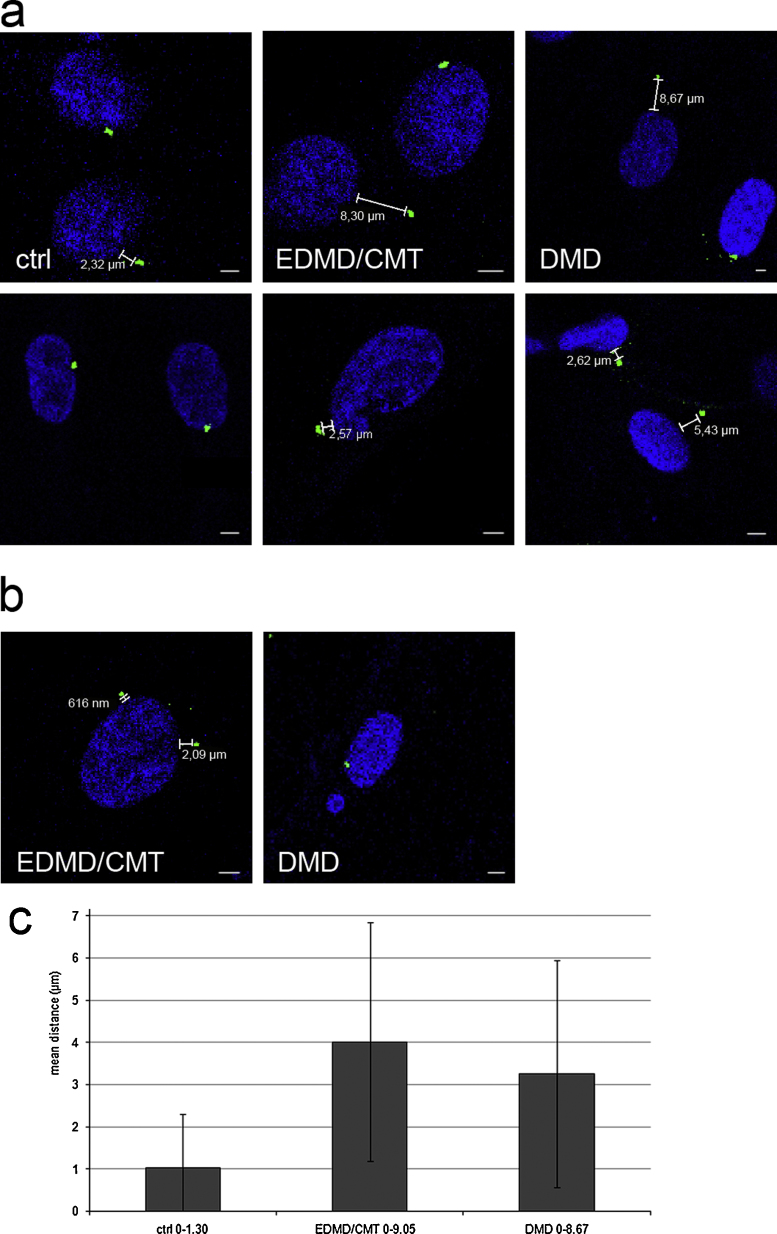
Nucleus-centrosome distance is altered in patient fibroblasts. (a, b) Immunofluorescence analysis of control (ctrl), EDMD/CMT and DMD fibroblasts using pericentrin specific antibodies to localise the centrosome. Detection was with Alexa Fluor 488 conjugated secondary antibodies, DAPI was used for nuclear staining. A, normal shaped nuclei, B, irregular shaped nuclei. Size bar, 5 μm. (c) Statistical evaluation of the nucleus centrosome distance. Nucleus-centrosome mean distances were measured in μm with Leica LAS AF Lite software. Fifty cells for control, EDMD/CMT, DMD fibroblasts each were examined. The error bars indicate standard deviations. For control cells we observed distances between 0 and 1.30 μm, for EDMD/CMT between 0 and 9.05 μm, and for DMD they were between 0 and 8.67 μm. Passage numbers were 17 (control cells), 6 (DMD) and 14 (EDMD/CMT).

The centrosome plays a key role in cellular architecture by determining the position of several associated organelles including the nucleus. Previous data indicated that nuclear envelope proteins like the LINC complex components and Emerin are participating in centrosome-nucleus juxtaposition and mediate shuttling of nuclear and centrosomal proteins between these organelles (Schneider et al., 2011; Salpingidou et al., 2007). Therefore we investigated the localisation of the centrosome relative to the nucleus using antibodies against pericentrin (Fig. 7a, b). In control cells all centrosomes were in close proximity of the nucleus (1.3 μm mean distance). Normal shaped as well as misshapen nuclei from patient cells exhibited a slightly increased centrosome-nucleus distance (Fig. 7b). The mean distance measured for DMD cells was 3.25 μm and for EDMD/CMT fibroblasts it had increased to 4 μm (Fig. 7c). The centrosome number per cell was not affected. In control as well as in patient cells the centrosome nucleus number correlated irrespective of the nuclear shape.

### Cell adhesion is altered in patient fibroblasts

**Fig. 8:**
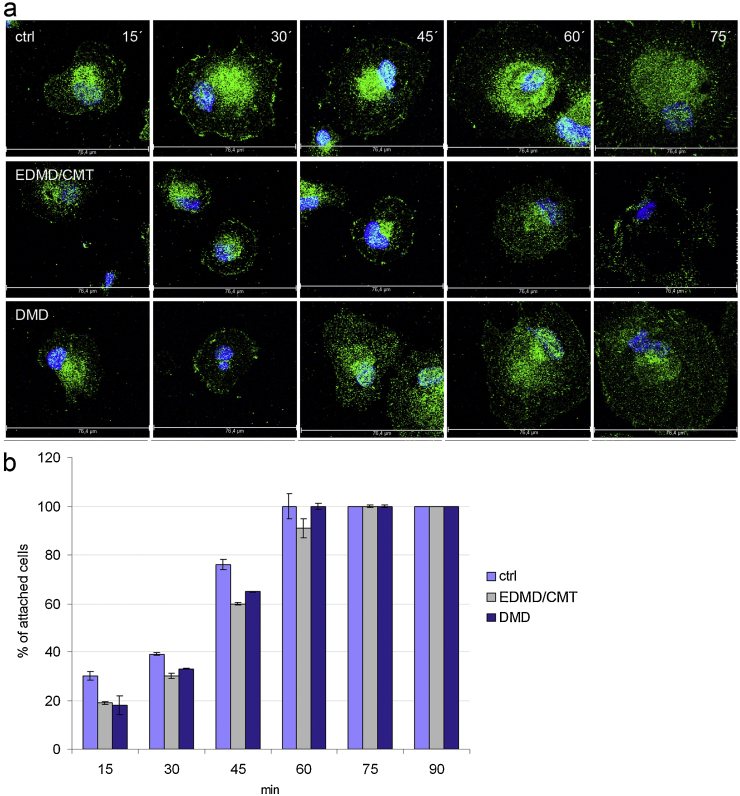
Cell surface adhesion in patient fibroblasts. (a) Immunofluorescence analysis of control and patient fibroblasts 15, 30, 45, 60 and 75 min after seeding. Staining was for Vinculin. Alexa Fluor 488 conjugated secondary antibody was used, DAPI for nuclear staining. Scale bar, 76.4 μm (shown in every panel). (b) Adhesion ability of control and patient fibroblasts 15, 30, 45, 60 and 75 min after seeding. At 90 min after seeding attachment was completed. The error bars indicate standard deviations.

Cell adhesion contributes substantially to the maintenance of tissue structure, the promotion of cell migration, and the transduction of information about the microenvironment of the cell. Vinculin is a plasma membrane-associated cytoskeletal protein in focal adhesion sites that is involved in the linkage of integrin adhesion molecules to the actin cytoskeleton (Ziegler et al., 2006). When we assessed cell adhesion by vinculin staining we found that all cell lines attached to the substratum and adhesion increased progressively as revealed by vinculin staining (Fig. 8a). Control cells had at every time point the largest area of spreading on the substratum. Notably, when settling of the cells was completed after 75 min, the spreading area of the patient cells was approximately two fold lower than that of the control cells (Table 2). A statistic evaluation of the adhesion ability showed that 30% of the control cells had attached to the coverslip after 15 min. Attachment to the substratum was significantly lower in EDMD/CMT and DMD cells at 30 and 45 min in comparison to the control cells. After 60 min control and DMD cells had attached completely and after 75 min EDMD/CMT cells had also completed attachment (Fig. 8b).

### Cell migration is altered in the mutant fibroblasts in an in vitro wound healing assay

**Fig. 9:**
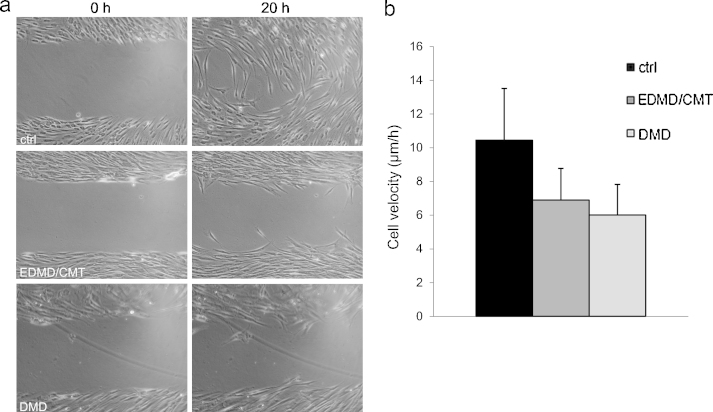
In vitro wound healing is altered in patient fibroblasts. (a) A cell migration assay was carried out to measure the wound healing capacity of patient fibroblasts. Confluent monolayers of control, EDMD/CMT and DMD fibroblasts were scratched and wound closure was monitored by bright field microscopy over 20 h. Representative images of each cell type at 0 h and 20 h are shown. (b) Graphical representation of the rate of migration. Each experiment was repeated at least thrice. The error bars indicate standard deviations.

Earlier studies have demonstrated that mutations in key NE proteins can result in slower migration rates of fibroblasts (Houben et al., 2009; Lee et al., 2007; Lüke et al., 2008). To investigate whether the mutations in the patient cells studied in this report also show similar defects we performed wound healing assays on confluent monolayers of patient fibroblasts. This assay allows to test the behaviour of the cells after physical injury of a cell layer and also to evaluate the migration behaviour and speed of the cells when they close the wound. Control fibroblasts quickly migrated into the wound exhibiting a cell velocity of 10.4 ± 3.07 μm per hour. Mutant fibroblasts oriented towards the wound, however the time required for wound closure was significantly increased and the cell velocities measured were reduced (6.9 ± 1.88 μm for EDMD/CMT and 6.0 ± 0.81 μm for DMD) (Fig. 9).

### Senescence is increased in patient fibroblasts

**Fig. 10:**
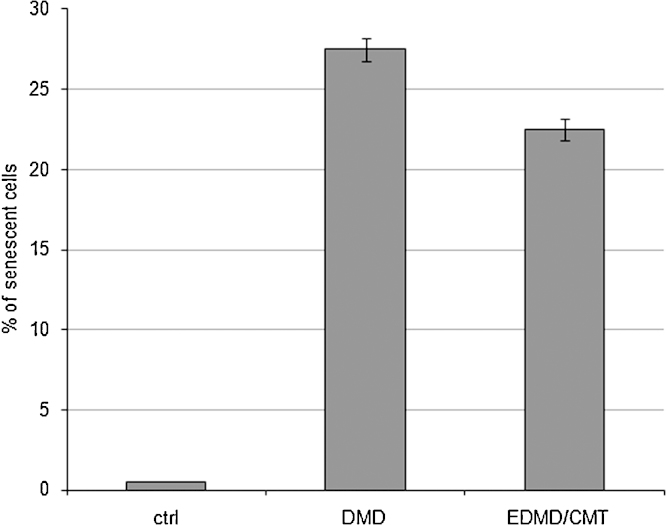
Statistical evaluation of expression of β-galactosidase as senescence marker. Control and patient fibroblasts were assayed for β-galactosidase expression. The experiment was done in duplicate, 200 cells were counted each. The error bars indicate standard deviations. Control fibroblasts were taken at passage 22, EDMD/CMT fibroblasts at passage 16, and DMD fibroblasts at passage 8.

An increased senescence has been reported for cells harbouring defects in components of the nuclear envelope (Le Dour et al., 2011). When we stained for the senescence marker β-galactosidase we found that in control fibroblasts of comparable passage numbers less than 1% of the cells were positive. In contrast, EDMD/CMT cells revealed a positive staining in 11% of the cells, and in DMD fibroblasts this number increased to 14% (Fig. 10).

### DMD fibroblasts display hypersensitivity to heat shock treatment

**Fig. 11:**
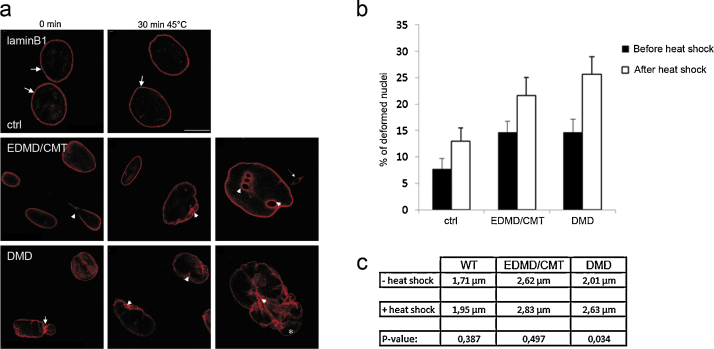
Heat shock induces severe nuclear shape alterations in DMD and EDMD/CMT fibroblasts. (a) Control and patient fibroblasts were subjected to heat shock treatment at 45 °C for 30 min and fixed immediately in ice cold methanol and immunolabelled for LaminB1. Untreated cells (left column) and heat stressed nuclei (middle and right columns) as indicated on top are shown. Arrows and arrowheads point to alterations. Bar, 10 μm. (b) The number of deformed nuclei prior to and after heat shock was determined. Cells were fixed and stained for Emerin or with DAPI to reveal the nuclear shape. (c) Analysis of the nucleus centrosome distance after heat shock. The cells were stained for Emerin and Pericentrin as centrosomal marker. The experiment was carried out at passage number 14 (control cells), 16 (DMD), 13 (EDMD/CMT). One hundred cells were analysed per strain.

It has been demonstrated that nuclei from laminopathy cells are susceptible to heat stress (Vigouroux et al., 2001). To evaluate the resistance of the nuclear envelope to damage induced by heat stress, control and patient fibroblasts were subjected to heat shock treatment for 30 min at 45 °C. Following the treatment, the cells were immediately fixed and stained for LaminB1 to assess nuclear shape changes. In control cells LaminB1 distribution and nuclear shape were not dramatically altered upon heat stress whereas the number of deformed nuclei was increased (Fig. 11a, b). Fibroblasts from DMD patients displayed profound nuclear abnormalities after treatment. Nuclear envelopes were deformed and disorganised into folds and pleats and many had a ruffled appearance. Many nuclei also showed indentations (Fig. 11a, small arrowhead), tears in the nuclear envelope and extensive lobulations (Fig. 11a, asterisk). Further, heavily deformed nuclei appeared enlarged in both patient cell lines suggesting an alteration in the nuclear protein network that resulted in hypersensitivity to heat stress induced deformation. In quantitative analysis we observed a higher number of deformed nuclei in untreated patient cells as compared to control cells. This number nearly doubled upon heat shock. A similar doubling was also seen in heat treated control cells (Fig. 11b).

We also determined whether heat shock affected the centrosome positioning and stained for the centrosome marker pericentrin. We found that the nucleosome–centrosome distance was slightly but not significantly increased in control and EDMD/CMT fibroblasts. In DMD fibroblasts the centrosome distance increased after heat shock. The difference was statistically significant (Fig. 11c).

## Discussion

Here we characterised dermal fibroblasts from patients suffering from DMD and EDMD/CMT. In case of patient G-11235 the disease causing mutation was located in the dystrophin gene, the mutation in patient G-11847 is not known. In addition to the disease causing mutations the patients carry heterozygous mutations in Nesprin-1α and in SUN1 (patient G-11235) and SUN2 (G-11847) which are located in the N-termini of the proteins. These mutations are normally not disease relevant, however, in combination with further mutations they may enhance the phenotype. Nesprin-1α has been characterised quite well. In particular, binding to Emerin and to LaminA/C have been described. Furthermore, it can homodimerise through its spectrin repeats 3 and 5 to form an antiparallel homodimer (Mislow et al., 2002b). Whether these repeats also interact with the related spectrin repeats of Nesprin-2 to form a heterodimer cannot be excluded as recent work showed that Nesprins can interact with each other and form a network surrounding the nucleus (Taranum et al., 2012). In addition, through its C-terminal KASH domain Nesprin-1α is a component of the LINC complex (Padmakumar et al., 2005). The mutations in the N-termini of SUN1 and SUN2, respectively, may contribute to the observed phenotypes as they may be able to interact with Nesprin-1 isoforms in analogy to the observed interaction with Nesprin-2 isoforms. They also have the potential to bind to LaminA/C (Haque et al., 2010) (see discussion below).

Our analysis of the patient fibroblast lines showed abnormalities in the nuclear structure with concomitant mislocalisation of Nesprins, Emerin, LaminA/C and LaminB1 at the NE, and alterations in adhesion, migration and further physiological properties such as senescence and stress sensitivity which are in general characteristic for fibroblasts from laminopathies.

In general, nuclear defects have not been studied for DMD cells because Dystrophin is a protein of the sarcolemma. Lack of the protein affects the mechanical integrity of myofibres and leaves them vulnerable to mechanical stress. Furthermore, aberrant activation of various signalling processes and increased apoptosis has been observed (Le Dour et al., 2011; Kumar and Boriek, 2003). The mechanisms are however not clear. If we consider the possibility that there is a continuous link between the nucleoplasm, the NE, the cytoskeleton and the plasma membrane, then Dystrophin at the sarcolemma can be integrated into this scenario. It is then also not surprising that the defects observed in DMD and laminopathies can overlap and resemble each other. At the cellular level we did however not note obvious nuclear defects in DMD myoblasts. The NE related changes we observed in the patient fibroblasts may therefore be a consequence of the additional mutations in the Nesprin-1 gene.

Interestingly, Nesprin-1 and Nesprin-2 are expressed in control and mutant fibroblasts and we observed alterations for both. Both proteins have a similar overall structure and have similar or identical binding partners (Simpson and Roberts, 2008). Whether the changes in Nesprin-1 and Nesprin-2 are due to a possible interaction between them or through alterations in the state of their common binding partners Emerin and LaminA/C is not clear. In case of the DMD patient cells we found a reduction in Nesprin-2 Giant at the NE in dysmorphic nuclei by immunofluorescence analysis although overall protein levels were not altered. Loss of Nesprin-2 has been shown to cause severe nuclear morphology defects in *LMNA* S143F progeria cells (Kandert et al., 2007) and Nesprin-2 knockout mice show an increase in nuclear volume (Lüke et al., 2008) suggesting that loss of Nesprin-2 Giant from the NE results in nuclear morphology defects. The observed mislocalisation of Nesprin-2 may therefore be a factor that contributes to the severity of the condition.

Increased fragility of the nucleus to heat shock has been reported in patients with Dunnigan type familial partial lipodystrophy (FPLD) as a functional consequence of an altered nuclear lamina structure (Le Dour et al., 2011). Similar analyses with our patient cells revealed that nuclei from both probands were hypersensitive to heat stress. Patient nuclei showed extensive changes with the appearance of blebs, pleats, folds, and indentations in the nuclei upon hyperthermic shock. Furthermore, enlargement of nuclei upon heat shock was observed in both patient samples. A subset of nuclei had a ruffled appearance and was extensively lobulated indicating diminished resistance to stress.

There is evidence that alterations in nuclear morphology correlate with chromatin rearrangement possibly involved in the control of gene expression (D’Angelo and Hetzer, 2006). These changes include altered sub-nuclear targeting of transcription factors and/or nuclear domains. Principal components of chromatin remodelling complexes include actin and actin regulatory proteins. Between nuclear actin, LaminA/C and Emerin a molecular link has been suggested (Clements et al., 2000; Fairley et al., 1999; Vaughan et al., 2001). Previously reported and further elucidated in the study by Haque et al. (2010), is a direct interaction of LaminA/C and the N-termini of SUN1 and SUN2. Noteworthy, both patients carry additional mutations in the N-termini of SUN1 (DMD) and SUN2 (EDMD/CMT), respectively. Loss or mutations that inhibit the interaction between one of those proteins could result in an altered relationship between the NE and chromatin. This can be extended to any protein that is a member of the LINC complex. Interestingly, putative interaction partners of SUN2 differ significantly as far as architectural proteins are concerned in EDMD/CMT cell lysates in comparison to control cell lysates. Actin and actin related proteins like α-actinin, myosin and Nesprin-2 were only detected in control lysates. Since LaminA/C was still present in EDMD/CMT cell lysates, it is conceivable that the LaminA/C-SUN2 interaction is maintained in EDMD/CMT cells, and the interruption might be therefore up- or downstream of the LaminA/C-SUN2 connection. In any case, the absence of the actin complex in the patient cell lysate points to an altered cytoskeletal interaction.

Spectrin repeat proteins are known to associate with F-actin not only through their actin binding domains but also through binding sites along the molecule (Djinovic-Carugo et al., 2002). In a hypothetical scenario, mutated Nesprin-1α in EDMD/CMT cells might weaken the actin complex interaction and, by doing so, contribute to the disease phenotype. It is therefore conceivable that altered expression of LINC complex proteins and/or associated proteins which interact with a nuclear actin scaffold may affect gene expression in repair and/or maintenance of muscle fibres in laminopathy patients. Further experiments assessing the interactions between LINC complex proteins and actin complex proteins will shed light on these questions.

The mutation in the dystrophin gene found in the DMD patient converts glutamic acid encoded by GAA/GAG into a premature termination codon resulting in a truncated protein which is lacking a significant part of its rod domain onto which many interactions have been mapped. This loss of protein function might disconnect the extra-cellular matrix from the cytoskeleton as these binding sites are lost. Additionally, the mutation found in Nesprin-1α2 in the DMD patient and in Nesprin-1α1 in the EDMD/CMT patient together with the SUN mutations being without consequences in healthy individuals when occurring separately, might contribute to weakened LINC complex protein interactions and thus perturb signaling.

## Figures and Tables

**Table 1:** Proteins in control and EDMD/CMT fibroblast lysates that are precipitated by GST-SUN2Nt. They belong to the following categories, gene regulation (A), RNA processing (B), architectural complex (C) and signalling (D). Identifier indicates the SwissProt accession number if not stated otherwise. Ctrl, control fibroblast lysate, EDMD/CMT, lysate from patient fibroblasts. +, presence; −, absence.

Identifier	Name	Category	Ctrl	EMD/CMT
Q9NVE4 (UniProtKB)	CCDC87	A	+	+
P68104 (UniProtKB)	EF1A1	B	−	+
NP_991247.1	HNRNPH1	B	+	+
O00571	mRNA helicase 3X	B	+	−
EEF2 HUMAN	EEF2	B	+	−
P60842 (UniProtKB)	eIF4A-1	B	+	+
Q8N9N8	EIF1AD	B	−	+
Q9UHB6	LIMA1	C	+	−
Q8WZ42	titin	C	−	+
O43707.2	α-Actinin1	C	+	−
P12814.2	α-Actinin4	C	+	−
P41351	Tubulin α	C	+	+
P05219	Tubulin β	C	+	+
Q9ULV4.1	Coronin1C	C	+	−
P02545.1	laminA/C*	C, D	+	+
P68032	Actin*	C, D	+	−
P35579	Myosin9*	C, D	+	−
O00159	Myosin1c*	C, D	+	−
Q8WXH0	Nesprin2*	C, D	+	−
P53618.3	Coatomer β	D	+	−
AAH20498.2	Coatomer γ	D	+	−
AAH20498.2	Coatomer γ	D	−	+
P22314	Ubiquitin-like modifier-activating enzyme	D	−	+

**Table 2:** Analysis of cell spreading in control and patient fibroblasts. The vinculin stained area on the surface of coverslips of control and patient fibroblasts 15, 30, 45, 60 and 75 min after seeding is measured in μm^2^.

	15 min	30 min	45 min	60 min	75 min
ctrl	1277.5	2385.51	2389.04	3882.86	7165.07
EDMD/CMT	978.02	936.09	1453.69	3058.94	3193.63
DMD	989.18	1126.05	1800.52	2691.37	4012.26

## References

[bib0005] Apel E.D., Lewis R.M., Grady R.M., Sanes J.R. (2000). Syne-1, a dystrophin- and Klarsicht-related protein associated with synaptic nuclei at the neuromuscular junction. J. Biol. Chem..

[bib0010] Bione S., Maestrini E., Rivella S., Mancini M., Regis S., Romeo G., Toniolo D. (1994). Identification of a novel X-linked gene responsible for Emery Dreifuss muscular dystrophy. Nat. Gen..

[bib0015] Bonne G., Di Barletta M.R., Varnous S., Becane H.M., Hammouda E.H., Merlini L., Muntoni F., Greenberg C.R., Gary F., Urtizberea J.A., Duboc D., Fardeau M., Toniolo D., Schwartz K. (1999). Mutations in the gene encoding lamin A/C cause autosomal dominant Emery Dreifuss muscular dystrophy. Nat. Gen..

[bib0020] Capell B.C., Collins F.S. (2006). Human laminopathies: nuclei gone genetically awry. Nat. Rev. Genet..

[bib0025] Clements L., Manilal S., Love D.R., Morris G.E. (2000). Direct interaction between emerin and lamin A. Biochem. Biophys. Res. Commun..

[bib0030] Crisp M., Liu Q., Roux K., Rattner J.B., Shanahan C., Burke B., Stahl P.D., Hodzic D. (2006). Coupling of the nucleus and cytoplasm: role of the LINC complex. J. Cell Biol..

[bib0035] D’Angelo M.A., Hetzer M.W. (2006). The role of the nuclear envelope in cellular organization. Cell. Mol. Life Sci..

[bib0040] Djinovic-Carugo K., Gautel M., Ylänne J., Young P. (2002). The spectrin repeat: a structural platform for cytoskeletal protein assemblies. FEBS Lett..

[bib0045] Fairley E.A., Kendrick-Jones J., Ellis J.A. (1999). The Emery-Dreifuss muscular dystrophy phenotype arises from aberrant targeting and binding of emerin at the inner nuclear membrane. J. Cell Sci..

[bib0050] Haque F., Lloyd D.J., Smallwood D.T., Dent C.L., Shanahan C.M., Fry A.M., Trembath R.C., Shackleton S. (2006). SUN1 interacts with nuclear lamin A and cytoplasmic nesprins to provide a physical connection between the nuclear lamina and the cytoskeleton. Mol. Cell Biol..

[bib0055] Haque F., Mazzeo D., Patel J.T., Smallwood D.T., Ellis J.A., Shanahan C.M., Shackleton S. (2010). Mammalian SUN protein interaction networks at the inner nuclear membrane and their role in laminopathy disease processes. J. Biol. Chem..

[bib0060] Houben F., Willems C.H., Declercq I.L., Hochstenbach K., Kamps M.A., Snoeckx L.H., Ramaekers F.C., Broers J.L. (2009). Disturbed nuclear orientation and cellular migration in A-type lamin deficient cells. Biochim. Biophys. Acta.

[bib0065] Kandert S., Lüke Y., Kleinhenz T., Neumann S., Lu W., Jaeger V.M., Munck M., Wehnert M., Müller C.M., Zhou Z., Noegel A.A., Dabauvalle M.C., Karakesisoglou I. (2007). Nesprin-2 Giant safeguards nuclear envelope architecture in LMNA S143F progeria cells. Hum. Mol. Gen..

[bib0070] Kumar A., Boriek A.M. (2003). Mechanical stress activates the nuclear factor-kappaB pathway in skeletal muscle fibers: a possible role in Duchenne muscular dystrophy. FASEB J..

[bib0075] Legrand B., Giudice E., Nicolas A., Delalande O., Le Rumeur E. (2011). Computational study of the human dystrophin repeats: interaction properties and molecular dynamics. PLoS One.

[bib0080] Le Dour C., Schneebeli S., Bakiri F., Darcel F., Jacquemont M.L., Maubert M.A., Auclair M., Jeziorowska D., Reznik Y., Béréziat V., Capeau J., Lascols O., Vigouroux C. (2011). A homozygous mutation of Prelamin-A preventing its farnesylation and maturation leads to a severe lipodystrophic phenotype: new insights into the pathogenicity of nonfarnesylated Prelamin-A. J. Clin. Endocrinol. Metab..

[bib0085] Lee J.S., Hale C.M., Panorchan P., Khatau S.B., George J.P., Tseng Y., Stewart C.L., Hodzic D., Wirtz D. (2007). Nuclear lamin A/C deficiency induces defects in cell mechanics, polarization, and migration. Biophys. J..

[bib0090] Libotte T., Zaim H., Abraham S., Padmakumar V.C., Schneider M., Lu W., Munck M., Hutchison C., Wehnert M., Fahrenkrog B., Sauder U., Aebi U., Noegel A.A., Karakesisoglou I. (2005). Lamin A/C-dependent localization of Nesprin-2, a giant scaffolder at the nuclear envelope. Mol. Biol. Cell.

[bib0095] Lu W., Gotzmann J., Sironi L., Jaeger V.M., Schneider M., Lüke Y., Uhlén M., Szigyarto C.A., Brachner A., Ellenberg J., Foisner R., Noegel A.A., Karakesisoglou I. (2008). Sun1 forms immobile macromolecular assemblies at the nuclear envelope. Biochim. Biophys. Acta.

[bib0100] Lüke Y., Zaim H., Karakesisoglou I., Jaeger V.M., Sellin L., Lu W., Schneider M., Neumann S., Beijer A., Munck M., Padmakumar V.C., Gloy J., Walz G., Noegel A.A. (2008). Nesprin-2 Giant (NUANCE) maintains nuclear envelope architecture and composition in skin. J. Cell Sci..

[bib0105] Manilal S., Nguyen T.M., Sewry C.A., Morris G.E. (1996). The Emery Dreifuss muscular dystrophy protein, emerin, is a nuclear membrane protein. Hum. Mol. Genet..

[bib0110] Mellad J.A., Warren D.T., Shanahan C.M. (2011). Nesprins LINC the nucleus and cytoskeleton. Curr. Opin. Cell Biol..

[bib0115] Mislow J.M., Kim M.S., Davis D.B., McNally E.M. (2002). Myne-1, a spectrin repeat transmembrane protein of the myocyte inner nuclear membrane, interacts with lamin A/C. J. Cell Sci..

[bib0120] Mislow J.M., Holaska J.M., Kim M.S., Lee K.K., Segura-Totten M., Wilson K.L., McNally E.M. (2002). Nesprin-1alpha self-associates and binds directly to emerin and lamin A in vitro. FEBS Lett..

[bib0125] Nagano A., Koga R., Ogawa M., Kurano Y., Kawada J., Okada R., Hayashi Y.K., Tsukahara T., Arahata K. (2006). Emerin deficiency at the nuclear membrane in patients with Emery Dreifuss muscular dystrophy. Nat. Gen..

[bib0130] Padmakumar V.C., Abraham S., Braune S., Noegel A.A., Tunggal B., Karakesisoglou I., Korenbaum E. (2004). Enaptin, a giant actin-binding protein, is an element of the nuclear membrane and the actin cytoskeleton. Exp. Cell Res..

[bib0135] Padmakumar V.C., Libotte T., Lu W., Zaim H., Abraham S., Noegel A.A., Gotzmann J., Foisner R., Karakesisoglou I. (2005). The inner nuclear membrane protein Sun1 mediates the anchorage of Nesprin-2 to the nuclear envelope. J. Cell Sci..

[bib0140] Simpson J.G., Roberts R.G. (2008). Patterns of evolutionary conservation in the nesprin genes highlight probable functionally important domains and isoforms. Biochem. Soc. Trans..

[bib0145] Salpingidou G., Smertenko A., Hausmanowa-Petrucewicz I., Hussey P.J., Hutchison C.J. (2007). A novel role for the nuclear membrane protein emerin in association of the centrosome to the outer nuclear membrane. J. Cell Biol..

[bib0150] Schneider M., Lu W., Neumann S., Brachner A., Gotzmann J., Noegel A.A., Karakesisoglou I. (2011). Molecular mechanisms of centrosome and cytoskeleton anchorage at the nuclear envelope. Cell. Mol. Life Sci..

[bib0155] Starr D.A., Fridolfsson H.N. (2010). Interactions between nuclei and the cytoskeleton are mediated by SUN-KASH nuclear-envelope bridges. Annu. Rev. Cell Dev. Biol..

[bib0160] Starr D.A., Hermann G.J., Malone C.J., Fixsen W., Priess J.R., Horvitz H.R., Han M. (2001). Unc-83 encodes a novel compartment of the nuclear envelope and is essential for proper nuclear migration. Development.

[bib0165] Starr D.A., Han M. (2002). Role of ANC-1 in tethering nuclei to the actincytoskeleton. Science.

[bib0170] Stewart C.L., Roux K.J., Burke B. (2007). Blurring the boundary: the nuclear envelope extends its reach. Science.

[bib0175] Stewart-Hutchinson P.J., Hale C.M., Wirtz D., Hodzic D. (2008). Structural requirements for the assembly of LINC complexes and their function in cellular mechanical stiffness. Exp. Cell Res..

[bib0180] Taranum, S., Sur, I., Müller, R., Lu, W., Rashmi, R.N., Munck, M., Neumann, S., Karakesisoglou, I., Noegel, A.A., 2012. Cytoskeletal interactions at the nuclear envelope mediated by Nesprins. Int. J. Cell Biol., doi:10.1155/2012/736524. 10.1155/2012/736524 PMC3296292 22518138

[bib0185] van der Loo B., Fenton M.J., Erusalimsky J.D. (1998). Cytochemical detection of a senescence-associated beta-galactosidase in endothelial and smooth muscle cells from human and rabbit blood vessels. Exp. Cell Res..

[bib0190] Vaughan A., Alvarez-Reyes M., Bridger J.M., Broers J.L., Ramaekers F.C., Wehnert M., Morris G.E., Whitfield W.G.F., Hutchison C.J. (2001). Both emerin and lamin C depend on lamin A for localization at the nuclear envelope. J. Cell Sci..

[bib0195] Vigouroux C., Auclair M., Dubosclard E., Pouchelet M., Capeau J., Courvain J., Buendia B. (2001). Nuclear envelope disorganization in fibroblasts from lipodystrophic patients with heterozygous R482Q/W mutations in the lamin A/C gene. J. Cell Sci..

[bib0200] Zhang Q., Bethmann C., Worth N.F., Davies J.D., Wasner C., Feuer A., Ragnauth C.D., Yi Q., Mellad J.A., Warren D.T., Wheeler M.A., Ellis J.A., Skepper J.N., Vorgerd M., Schlotter-Weigel B., Weissberg P.L., Roberts R.G., Wehnert M., Shanahan C. (2007). Nesprin-1 and -2 are involved in the pathogenesis of Emery Dreifuss muscular dystrophy and are critical for nuclear envelope integrity. Hum. Mol. Gen..

[bib0205] Zhen Y.Y., Libotte T., Munck M., Noegel A.A., Korenbaum E. (2002). NUANCE, a giant protein connecting the nucleus and actin cytoskeleton. J. Cell Sci..

[bib0210] Ziegler W.H., Liddington R.C., Critchley D.R. (2006). The structure and regulation of vinculin. Trends Cell Biol..

