# Supplementary material for: LINC complex alterations in DMD and EDMD/CMT fibroblasts
Source: Eur J Cell Biol. 2012 Aug;91(8):614–28. doi: 10.1016/j.ejcb.2012.03.003 (PMC3778752; doi:10.1016/j.ejcb.2012.03.003)
Supplement: Supplementary file 1 [file mmc1.doc]

Ms. Ref. No.: EJCB-D-11-00190
Title: LINC complex alterations in DMD and EDMD/CMT fibroblasts
European Journal of Cell Biology

| Received | Nov 24, 2011 |
| --- | --- |
| Decision | Dec 21, 2011 |
| Revision received | Mar 08, 2012 |
| Accepted | Mar 09, 2012 |

**Decision letter**

Dear Angelika,

Your manuscript has now been seen by two expert referees. Both find high merit in your paper, but also raise some points that preclude publication in the current form. These include, among others, immunoprecipitation/pull down experiments to probe the disruption of the nesprin/SUN complex by overexpression of mutants or quantification of misshaped nuclei due to heatshock. Please try to address the referees´ points as closely as possible. We are looking forward to the revised version of your manuscript.

Please submit a list of changes or a rebuttal against each point which is being raised when you submit the revised manuscript.

Best regards
Stefan

Stefan Linder
Editor

p.p. Dagmar Gebauer
Editorial Office
European Journal of Cell Biology

Reviewers' comments:

Reviewer 1

This is a very carefully performed study on cells of two patients suffering under DMD respectively under EDMD/CMT. The authors have thoroughly   analyzed all relevant genes and could identify point mutations in Nesprin 1<alpha>, SUN1 and SUN2. Their studies clearly demonstrate that these mutations not only affect nuclear structure but also the cytoskeletal architecture including centrosome localization, cell mobility as well as cell adhesion and cell spreading.  The quantitative data presented indicate a reduction of SUN2 binding partners in diseased cells.  
Very interesting is the reaction of diseased cells to heat stress.  This is the only point where I ask the authors to include additional data if possible:
1.     Does the number of misshapen nuclei in diseased cells increase within the 30 minutes during heat shock? Please include quantitative data comparable to that shown in Fig. 1

2.     Does the heat shock also affect the centrosome position?  Please comment.
This is an excellent manuscript that needs only very minor revision prior to acceptance.  The data are of general interest for scientists interesting in cell architecture and cell function.

Reviewer 2
This study identified mutations in nesprin-1 and SUN1/2 genes of the LINC complex that connects the nucleus and the cytoskeleton and established a SUN2 proteome for control and patient fibroblasts, which provides additional information in further understanding the roles of both nesprin and SUN in EDMD.   The results generated from the current study are convincing, but more descriptive.  There are some biochemical experiments required to be carried out to further validate if these mutations play potential roles in contributing to all the observed changes in the cells:  1) Overexpression of the WT and mutants for nesprin-1alpha and/or SUN1/SUN2 and then in vitro IP or GST pull down to be performed to investigate if these mutations disrupt the nesprin/SUN/lamin/emerin binding. 2) qPCR for nesprin-1alpha2 to be carried out to study if the mutation affects this nesprin-1 isoform at the mRNA level. These will help to clarify if these mutations may contribute to disrupt/weaken the LINC complex and consequently perturb signalling, causing the disease phenotype.

---------------------------------------------------------------------------------------------------------------------------------------------------------

**Authors response letter**

Dear Stefan,

Thank you very much for considering our manuscript “LINC complex alterations in DMD and EDMD/CMT fibroblasts” for publication in the European Journal of Cell Biology and for inviting us to submit a revised version. We are most grateful for both your editorial comments and those of the referees, which helped us to improve the manuscript. The paper has been revised to address the points raised as detailed below.

Point-by-point response to points raised:

Reviewer 1

*….Very interesting is the reaction of diseased cells to heat stress. This is the only point where I ask the authors to include additional data if possible:*

*1. Does the number of misshapen nuclei in diseased cells increase within the 30 minutes during heat shock? Please include quantitative data comparable to that shown in Fig. 1*

We have carried out this experiment and determined the number of misshapen nuclei after heat shock and found that the number is increased. However, the nuclei of control cells are likewise affected. The data is shown in new Figure 11b and discussed on page 20, bottom and page 21, top.

*2. Does the heat shock also affect the centrosome position? Please comment.*

We thank the reviewer for this question. We determined the centrosome position after heat shock and found that it is slightly but not significantly increased after a heat shock in control and EDMD/CMT fibroblasts, whereas in DMD we found a statistically significant increase. The data is shown in new Figure 11c and discussed on page 21, top.

Reviewer 2

….*The results generated from the current study are convincing, but more descriptive. There are some biochemical experiments required to be carried out to further validate if these mutations play potential roles in contributing to all the observed changes in the cells: 1) Overexpression of the WT and mutants for nesprin-1alpha and/or SUN1/SUN2 and then in vitro IP or GST pull down to be performed to investigate if these mutations disrupt the nesprin/SUN/lamin/emerin binding.*

We agree with the reviewer that this is an important question. We generated the corresponding mutations and expressed GFP- and V5-tagged mutated SUN1 and SUN2 in HaCaT cells. We analyzed the cells at the immunofluorescence level and did not observe obvious alterations with respect to the distribution and abundance of nuclear envelope proteins. The rather low transfection efficiency however prevented further studies such as IP or pull down studies. A paragraph has been added (page 17, last paragraph) and a figure showing the results is included (new Figure 6a and b).

*2) qPCR for nesprin-1alpha2 to be carried out to study if the mutation affects this nesprin-1 isoform at the mRNA level. These will help to clarify if these mutations may contribute to disrupt/weaken the LINC complex and consequently perturb signalling, causing the disease phenotype.*

We feel that the proposed PCR analysis might give ambiguous results with regard to the Nesprin-1 isoforms as all smaller isoforms are contained within the larger ones (Simpson and Roberts, 2008). At the protein level we detected with polyclonal antibodies directed against the C-terminus primarily larger isoforms (Figure 1c). The presence of smaller one can however not be excluded.

Finally, we would like to thank you and the referees for your critical appraisal of the paper. We feel that, in addressing the points raised, we have significantly improved the accessibility and quality of the manuscript.

Kind regards,

Angelika
